# Event-driven contrastive divergence for spiking neuromorphic systems

**DOI:** 10.3389/fnins.2013.00272

**Published:** 2014-01-30

**Authors:** Emre Neftci, Srinjoy Das, Bruno Pedroni, Kenneth Kreutz-Delgado, Gert Cauwenberghs

**Affiliations:** ^1^Institute for Neural Computation, University of CaliforniaSan Diego, La Jolla, CA, USA; ^2^Electrical and Computer Engineering Department, University of CaliforniaSan Diego, La Jolla, CA, USA; ^3^Department of Bioengineering, University of CaliforniaSan Diego, La Jolla, CA, USA

**Keywords:** synaptic plasticity, neuromorphic cognition, Markov chain monte carlo, recurrent neural network, generative model

## Abstract

Restricted Boltzmann Machines (RBMs) and Deep Belief Networks have been demonstrated to perform efficiently in a variety of applications, such as dimensionality reduction, feature learning, and classification. Their implementation on neuromorphic hardware platforms emulating large-scale networks of spiking neurons can have significant advantages from the perspectives of scalability, power dissipation and real-time interfacing with the environment. However, the traditional RBM architecture and the commonly used training algorithm known as Contrastive Divergence (CD) are based on discrete updates and exact arithmetics which do not directly map onto a dynamical neural substrate. Here, we present an event-driven variation of CD to train a RBM constructed with Integrate & Fire (I&F) neurons, that is constrained by the limitations of existing and near future neuromorphic hardware platforms. Our strategy is based on neural sampling, which allows us to synthesize a spiking neural network that samples from a target Boltzmann distribution. The recurrent activity of the network replaces the discrete steps of the CD algorithm, while Spike Time Dependent Plasticity (STDP) carries out the weight updates in an online, asynchronous fashion. We demonstrate our approach by training an RBM composed of leaky I&F neurons with STDP synapses to learn a generative model of the MNIST hand-written digit dataset, and by testing it in recognition, generation and cue integration tasks. Our results contribute to a machine learning-driven approach for synthesizing networks of spiking neurons capable of carrying out practical, high-level functionality.

## 1. Introduction

Machine learning algorithms based on stochastic neural network models such as RBMs and deep networks are currently the state-of-the-art in several practical tasks (Hinton and Salakhutdinov, 2006; Bengio, 2009). The training of these models requires significant computational resources, and is often carried out using power-hungry hardware such as large clusters (Le et al., 2011) or graphics processing units (Bergstra et al., 2010). Their implementation in dedicated hardware platforms can therefore be very appealing from the perspectives of power dissipation and of scalability.

Neuromorphic Very Large Scale Integration (VLSI) systems exploit the physics of the device to emulate very densely the performance of biological neurons in a real-time fashion, while dissipating very low power (Mead, 1989; Indiveri et al., 2011). The distributed structure of RBMs suggests that neuromorphic VLSI circuits and systems can become ideal candidates for such a platform. Furthermore, the communication between neuromorphic components is often mediated using asynchronous address-events (Deiss et al., 1998) enabling them to be interfaced with event-based sensors (Liu and Delbruck, 2010; Neftci et al., 2013; O'Connor et al., 2013) for embedded applications, and to be implemented in a very scalable fashion (Silver et al., 2007; Joshi et al., 2010; Schemmel et al., 2010).

Currently, RBMs and the algorithms used to train them are designed to operate efficiently on digital processors, using batch, discrete-time, iterative updates based on exact arithmetic calculations. However, unlike digital processors, neuromorphic systems compute through the continuous-time dynamics of their components, which are typically Integrate & Fire (I&F) neurons (Indiveri et al., 2011), rendering the transfer of such algorithms on such platforms a non-trivial task. We propose here a method to construct RBMs using I&F neuron models and to train them using an online, event-driven adaptation of the (CD) algorithm.

We take inspiration from computational neuroscience to identify an efficient neural mechanism for sampling from the underlying probability distribution of the RBM. Neuroscientists argue that brains deal with uncertainty in their environments by encoding and combining probabilities optimally (Doya et al., 2006), and that such computations are at the core of cognitive function (Griffiths et al., 2010). While many mechanistic theories of how the brain might achieve this exist, a recent *neural sampling* theory postulates that the spiking activity of the neurons encodes samples of an underlying probability distribution (Fiser et al., (2010). The advantage for a neural substrate in using such a strategy over the alternative one, in which neurons encode probabilities, is that it requires exponentially fewer neurons. Furthermore, abstract model neurons consistent with the behavior of biological neurons can implement Markov Chain Monte Carlo (MCMC) sampling (Buesing et al., 2011), and RBMs sampled in this way can be efficiently trained using CD, with almost no loss in performance (Pedroni et al., 2013). We identify the conditions under which a dynamical system consisting of I&F neurons performs neural sampling. These conditions are compatible with neuromorphic implementations of I&F neurons (Indiveri et al., 2011), suggesting that they can achieve similar performance. The calibration procedure necessary for configuring the parameters of the spiking neural network is based on firing rate measurements, and so is easy to realize in software and in hardware platforms.

In standard CD, weight updates are computed on the basis of alternating, feed-forward propagation of activities (Hinton, 2002). In a neuromorphic implementation, this translates to reprogramming the network connections and resetting its state variables at every step of the training. As a consequence, it requires two distinct dynamical systems: one for normal operation (i.e., testing), the other for training, which is highly impractical. To overcome this problem, we train the neural RBMs using an online adaptation of CD. We exploit the recurrent structure of the network to mimic the discrete “construction” and “reconstruction” steps of CD in a spike-driven fashion, and Spike Time Dependent Plasticity (STDP) to carry out the weight updates. Each sample (spike) of each random variable (neuron) causes synaptic weights to be updated. We show that, over longer periods, these microscopic updates behave like a macroscopic CD weight update. Compared to standard CD, no additional connectivity programming overhead is required during the training steps, and both testing and training take place in the same dynamical system.

Because RBMs are generative models, they can act simultaneously as classifiers, content-addressable memories, and carry out probabilistic inference. We demonstrate these features in a MNIST hand-written digit task (LeCun et al., 1998), using an RBM network consisting of one layer of 824 “visible” neurons and one layer of 500 “hidden” neurons. The spiking neural network was able to learn a generative model capable of recognition performances with accuracies up to 91.9%, which is close to the performance obtained using standard CD and Gibbs sampling, 93.6%.

## 2. Materials and methods

### 2.1. Neural sampling with noisy I&F neurons

We describe here conditions under which a dynamical system composed of I&F neurons can perform neural sampling. It has been proven that abstract neuron models consistent with the behavior of biological spiking neurons can perform MCMC sampling of a Boltzmann distribution (Buesing et al., 2011). Two conditions are sufficient for this. First, the instantaneous firing rate of the neuron verifies:

(1) $$\rho (u(t),\;t-t^{\prime })=\{\begin{array}{ll} 0 & \text{if }t-t^{\prime }<\tau_{r}\\ r(u(t)) & t-t^{\prime }\geq \tau_{r} \end{array},$$

with *r*(*u*(*t*)) proportional to exp(*u*(*t*)), where *u*(*t*) is the membrane potential and τ_r_ is an absolute refractory period during which the neuron cannot fire. ρ(*u*(*t*), *t* − *t*′) describes the neuron's instantaneous firing rate as a function of *u*(*t*) at time *t*, given that the last spike occurred at *t*′. It can be shown that the average firing rate of this neuron model for stationary *u*(*t*) is the sigmoid function:

(2) $$\rho (u)={\left(\tau_{r}+\exp(-u)\right)}^{-1}.$$

Second, the membrane potential of neuron *i* is equal to the linear sum of its inputs:

(3) $$u_{i}(t)=b_{i}+\sum_{j\;=\;1}^{N}w_{ij}z_{j}(t),\forall i=1,\ldots ,N,$$

where *b*_*i*_ is a constant bias, and *z*_*j*_(*t*) represents the pre-synaptic spike train produced by neuron *j* defined as being equal to 1 when the pre-synaptic neuron spikes for a duration τ_*r*_, and equal to zero otherwise. The terms *w*_*ij*_*z*_*j*_(*t*) are identified with the time course of the Post–Synaptic Potential (PSP), i.e., the response of the membrane potential to a pre-synaptic spike. The two conditions above define a neuron model, to which we refer as the “abstract neuron model.” Assuming the network states are binary vectors [*z*_1_, …, *z*_*k*_], it can be shown that, after an initial transient, the sequence of network states can be interpreted as MCMC samples of the Boltzmann distribution:

(4) $$\begin{array}{l} p(z_{1},\ldots ,z_{k})=\frac{1}{Z}\exp\text{​}(-E(z_{1},\ldots ,z_{k})),\text{with}\\ E(z_{1},\ldots ,z_{k})=-\frac{1}{2}\sum_{ij}W_{ij}z_{i}z_{j}-\sum_{i}b_{i}z_{i}, \end{array}$$

where *Z* = ∑_*z*_1_, …, *z*_*k*__ exp (− *E*(*z*_1_, …, *z*_*k*_)) is a constant such that *p* sums up to unity, and *E*(*z*_1_, …, *z*_*k*_) can be interpreted as an energy function (Haykin, 1998).

An important fact of the abstract neuron model is that, according to the dynamics of *z*_*j*_(*t*), the PSPs are “rectangular” and non-additive since no two presynaptic spikes can occur faster than the refractive period. The implementation of synapses producing such PSPs on a large scale is very difficult to realize in hardware, when compared to first-order linear filters that result in “alpha”-shaped PSPs (Destexhe et al., 1998; Bartolozzi and Indiveri, 2007). This is because, in the latter model, the synaptic dynamics are linear, such that a single hardware synapse can be used to generate the same current that would be generated by an arbitrary number of synapses (see also next section). As a consequence, we will use alpha-shaped PSPs instead of rectangular PSPs in our models. The use of the alpha PSP over the rectangular PSP is the major source of degradation in sampling performance, as we will discuss in 2.2.

#### 2.1.1. Stochastic I&F neurons

A neuron whose instantaneous firing rate is consistent with Equation (1) can perform neural sampling. Equation (1) is a generalization of the Poisson process to the case when the firing probability depends on the time of the last spike (i.e., it is a renewal process), and so can be verified only if the neuron fires stochastically (Cox, 1962). Stochasticity in I&F neurons can be obtained through several mechanisms, such as a noisy reset potential, noisy firing threshold, or noise injection (Plesser and Gerstner, 2000). The first two mechanisms necessitate stochasticity in the neuron's parameters, and therefore may require specialized circuitry. But noise injection in the form of background Poisson spike trains requires only synapse circuits, which are present in many neuromorphic VLSI implementation of spiking neurons (Bartolozzi and Indiveri, 2007; Indiveri et al., 2011). Furthermore, Poisson spike trains can be generated self-consistently in balanced excitatory-inhibitory networks (van Vreeswijk and Sompolinsky, 1996), or using finite-size effects and neural mismatch (Amit and Brunel, 1997).

We show that the abstract neuron model in Equation (1) can be realized in a simple dynamical system consisting of leaky I&F neurons with noisy currents. The neuron's membrane potential below firing threshold θ is governed by the following differential equation:

(5) $$C\frac{\text{d}}{\text{d}t}u_{i}=-g_{L}u_{i}+I_{i}(t)+\sigma \xi (t),\;u_{i}(t)\in (-\infty ,\theta ),$$

where *C* is a membrane capacitance, *u*_*i*_ is the membrane potential of neuron *i*, *g*_*L*_ is a leak conductance, σ ξ(*t*) is a white noise term of amplitude σ (which can for example be generated by background activity), *I*_*i*_(*t*) its synaptic current and θ is the neuron's firing threshold. When the membrane potential reaches θ, an action potential is elicited. After a spike is generated, the membrane potential is clamped to the reset potential *u*_rst_ for a refractory period τ_*r*_.

In the case of the neural RBM, the currents *I*_*i*_(*t*) depend on the layer the neuron is situated in. For a neuron *i* in layer υ

(6) $$\begin{array}{l} I_{i}(t)=I_{i}^{d}(t)+I_{i}^{\upsilon }(t),\\ \tau_{\text{syn}}\frac{\text{d}}{\text{d}t}I_{i}^{\;\upsilon }=-I_{i}^{\;\upsilon }+\sum_{j\;=\;1}^{N_{h}}q_{h_{ji}}h_{j}(t)+q_{b_{i}}b_{\;\upsilon_{i}}(t), \end{array}$$

where *I*^*d*^_*i*_(*t*) is a current representing the data (i.e., the external input), *I*^υ^ is the feedback from the hidden layer activity and the bias, and the *q*'s are the respective synaptic weights, and *b*_υ_(*t*) is a Poisson spike train implementing the bias. Spike trains are represented by a sum of Dirac delta pulses centered on the respective spike times:

(7) $$b_{\;\upsilon_{i}}(t)=\sum_{k\;\in \;Sp_{i}}\delta (t-t_{k}),\;h_{j}(t)=\sum_{k\;\in \;Sp_{j}}\delta (t-t_{k})$$

where *Sp*_*i*_ and *Sp*_*j*_ are the set of the spike times of the bias neuron *b*_υ_*i*__ and the hidden neuron *h*_*j*_, respectively, and δ(*t*) = 1 if *t* = 0 and 0 otherwise.

For a neuron *j* in layer *h*,

(8) $$\begin{array}{l} I_{j}(t)=I_{j}^{h}(t),\\ \tau_{\text{syn}}\frac{\text{d}}{\text{d}t}I_{j}^{h}=-I_{j}^{h}+\sum_{i\;=\;1}^{N_{\upsilon }}q_{\;\upsilon_{ij}}\;\upsilon_{i}(t)+q_{b_{j}}b_{h_{j}}(t), \end{array}$$

where *I*^*h*^ is the feedback from the visible layer, and υ(*t*) and *b*_*h*_(*t*) are Poisson spike trains of the visible neurons and the bias neurons, defined similarly as in Equation (7). The dynamics of *I*^*h*^ and *I*^υ^ correspond to a first-order linear filter, so each incoming spike results in PSPs that rise and decay exponentially (i.e., alpha-PSP) (Gerstner and Kistler, 2002).

Can this neuron verify the conditions required for neural sampling? The membrane potential is already assumed to be equal to the sum of the PSPs as required by neural sampling. So to answer the above question we only need to verify whether Equation (1) holds. Equation (5) is a Langevin equation which can be analyzed using the Fokker–Planck equation (Gardiner, 2012). The solution to this equation provides the neuron's input/output response, i.e., its transfer curve (for a review, see Renart et al., 2003):

(9) $$\rho (u_{0})={\left(\tau_{r}+\tau_{m}\sqrt{\pi }\int_{\frac{u_{\text{rst}}-u_{0}}{\sigma_{V}}}^{\frac{\theta -u_{0}}{\sigma_{V}}}\text{d}x\exp(x^{2})(1+\text{erf}(x))\right)}^{-1},$$

where erf is the error function (the integral of the normal distribution), $u_{0}=\frac{I}{g_{L}}$ is the stationary value of the membrane potential when injected with a constant current *I*, $\tau_{m}=\frac{C}{g_{L}}$ is the membrane time constant, *u*_rst_ is the reset voltage, and σ^2^_V_(*u*) = σ^2^/(*g*_*L*_*C*).

**Figure 1:**
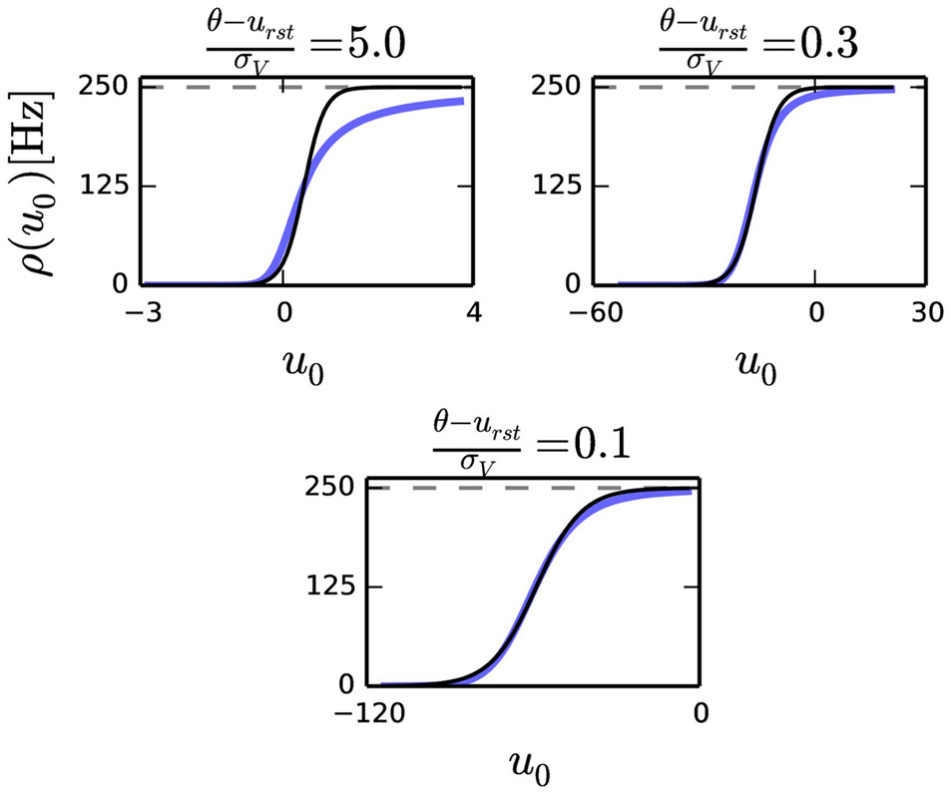
**Transfer curve of a leaky I&F neuron for three different parameter sets where $u_{0}=\frac{I}{g_{L}}$, and $\frac{1}{\tau_{r}}=250$ [Hz] (dashed gray)**. In this plot, σ_*V*_ is varied to produce different ratios $\frac{\theta -u_{\text{rst}}}{\sigma_{V}}$. The three plots above shows that the fit with the sigmoid function (solid black) improves as the ratio decreases.

According to Equation (2), the condition for neural sampling requires that the average firing rate of the neuron to be the sigmoid function. Although the transfer curve of the noisy I&F neuron Equation (9) is not identical to the sigmoid function, it was previously shown that with an appropriate choice of parameters, the shape of this curve can be very similar to it (Merolla et al., 2010). We observe that, for a given refractory period τ_*r*_, the smaller the ratio $\frac{\theta -u_{\text{rst}}}{\sigma_{V}}$ in Equation (5), the better the transfer curve resembles a sigmoid function (Figure 1). With a small $\frac{\theta -u_{\text{rst}}}{\sigma_{V}}$, the transfer function of a neuron can be fitted to

(10) $$\nu (I)=\frac{1}{\tau_{r}}{\left(1+\frac{\exp(-I\beta )}{\gamma \tau_{r}}\right)}^{-1},$$

where β and γ are the parameters to be fitted. The choice of the neuron model described in Equation (5) is not critical for neural sampling: A relationship that is qualitatively similar to Equation (9) holds for neurons with a rigid (reflective) lower boundary (Fusi and Mattia, 1999) which is common in VLSI neurons, and for I&F neurons with conductance-based synapses (Petrovici et al., 2013).

This result also shows that synaptic weights *q*_υ__*i*_, *qh*_*j*_, which have the units of charge are related to the RBM weights *W*_*ij*_ by a factor β^−1^. To relate the neural activity to the Boltzmann distribution, Equation (4), each neuron is associated to a binary random variable which is assumed to take the value 1 for a duration τ_*r*_ after the neuron has spiked, and zero otherwise, similarly to Buesing et al. (2011). With this encoding, the network state is characterized by a binary vector having the same number of entries as the number of neurons in the network. The relationship between this random vector and the I&F neurons' spiking activity is illustrated in Figure 3. The membrane potential of the neuron (black) evolves in a random fashion until it spikes, after which it is clamped to *u*_rst_ for a duration τ_*r*_ (gray). While the neuron is in the refractory period, the random variable associated to it is assumed to takes the value 1. This way, the state of the network can always be associated with a binary vector. According to the theory, the dynamics in the network guarantees that the binary vectors are samples drawn from a Boltzmann distribution.

#### 2.1.2. Calibration protocol

**Figure 2:**
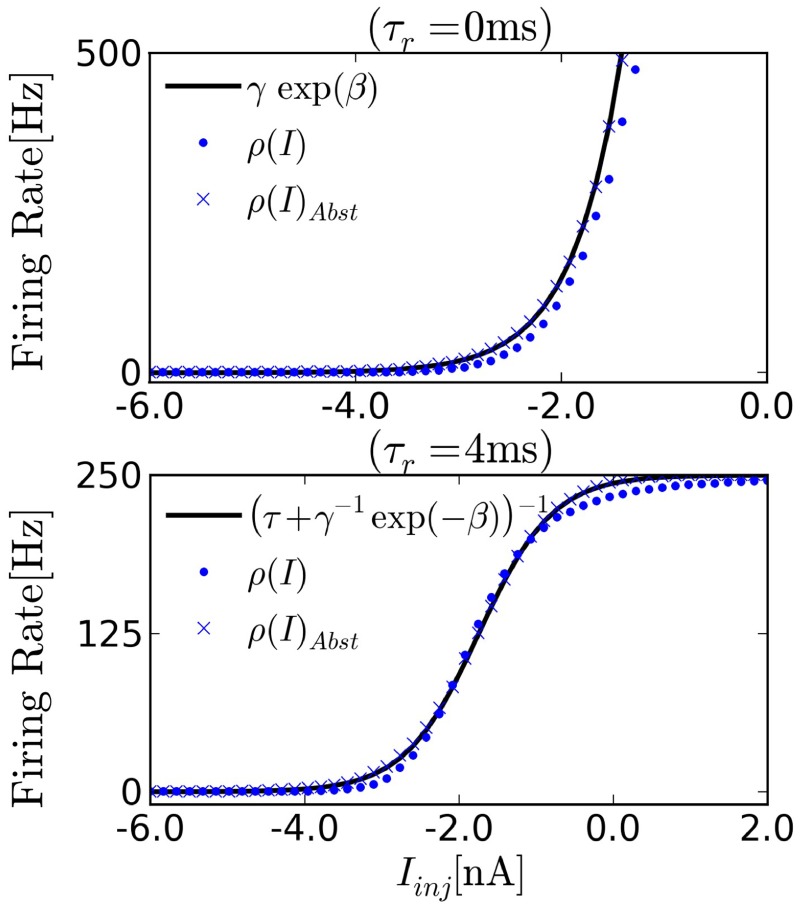
**Transfer function of I&F neurons driven by background white noise Equation (5)**. We measure the firing rate of the neuron as a function of a constant current injection to estimate ρ(*u*_0_), where for constant *I*_inj_, *u*_0_ = *I*_inj_/*g*_*L*_. (Top) The transfer function of noisy I&F neurons in the absence of refractory period [ρ(*u*) = *r*(*u*), circles]. We observe that ρ is approximately exponential over a wide range of inputs, and therefore compatible with neural sampling. Crosses show the transfer curve of neurons implementing the abstract neuron Equation (1), exactly. (Bottom) With an absolute refractory period the transfer function approximates the sigmoid function. The firing rate saturates at [250]Hz due to the refractory period chosen for the neuron.

In order to transfer the parameters from the probability distribution Equation (4) to those of the I&F neurons, the parameters γ, β in Equation (10) need to be fitted. An estimate of a neuron's transfer function can be obtained by computing its spike rate when injected with different values of constant inputs *I*. The refractory period τ_*r*_ is the inverse of the maximum firing rate of the neuron, so it can be easily measured by measuring the spike rate for very high input current *I*. Once τ_*r*_ is known, the parameter estimation can be cast into a simple linear regression problem by fitting log(ρ(*i*)^−1^ − τ_*r*_) with β*I* + log(γ). Figure 2 shows the transfer curve when τ_*r*_ = 0 ms, which is approximately exponential in agreement with Equation (1).

The shape of the transfer curse is strongly dependent on the noise amplitude. In the absence of noise, the transfer curve is a sharp threshold function, which softens as the amplitude of the noise is increased (Figure 1). As a result, both parameters γ and β are dependent on the variance of the input currents from other neurons *I*(*t*). Since β*q* = *w*, the effect of the fluctuations on the network is similar to scaling the synaptic weights and the biases which can be problematic. However, by selecting a large enough noise amplitude σ and a slow enough input synapse time constant, the fluctuations due to the background input are much larger than the fluctuations due to the inputs. In this case, β and γ remain approximately constant during the sampling.

Neural mismatch can cause β and γ to differ from neuron to neuron. From Equation (10) and the linearity of the postsynaptic currents *I*(*t*) in the weights, it is clear that this type of mismatch can be compensated by scaling the synaptic weights and biases accordingly. The calibration of the parameters γ and β quantitatively relate the spiking neural network's parameters to the RBM. In practice, this calibration step is only necessary for mapping pre-trained parameters of the RBM onto the spiking neural network.

Although we estimated the parameters of software simulated I&F neurons, parameter estimation based on firing rate measurements were shown to be an accurate and reliable method for VLSI I&F neurons as well (Neftci et al., 2012).

### 2.2. Validation of neural sampling using I&F neurons

**Figure 3:**
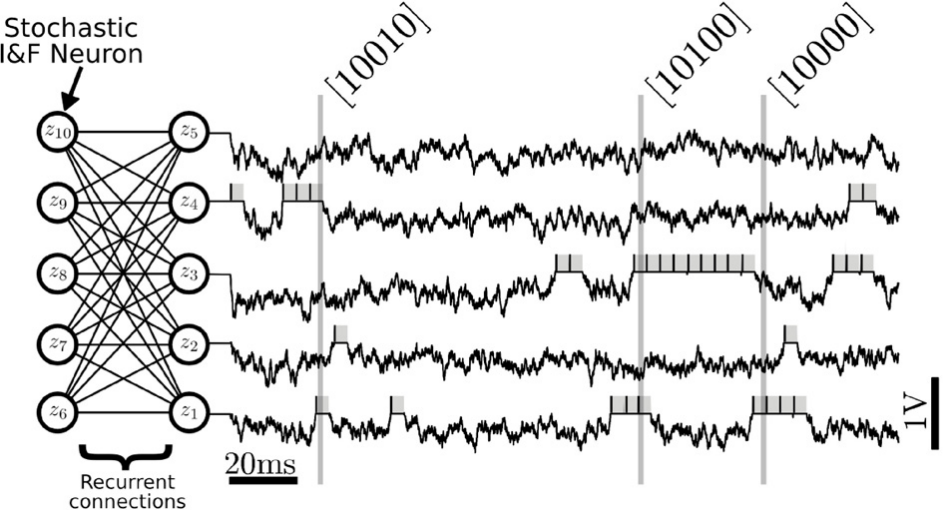
**Neural Sampling in an RBM consisting of 10 stochastic I&F neurons, with five neurons in each layer**. Each neuron is associated to a binary random variable which take values 1 during a refractory period τ_*r*_ after the neuron has spiked (gray shadings). The variables are sampled at 1 kHz to produce binary vectors that correspond to samples of the joint distribution *p*(*z*). In this figure, only the membrane potential and the samples produced by the first five neurons are shown. The vectors inside the brackets are example samples of the marginalized distribution *p*(*z*_1_, *z*_2_, *z*_3_, *z*_4_, *z*_5_) produced at the time indicated by the vertical lines. In the RBM, there are no recurrent connections within a layer.

The I&F neuron verifies Equation (1) only approximately, and the PSP model is different from the one of Equation (3). Therefore, the following two important questions naturally arise: how accurately does the I&F neuron-based sampler outlined above sample from a target Boltzmann distribution? How well does it perform in comparison to an exact sampler, such as the Gibbs sampler? To answer these questions we sample from several neural RBM consisting of five visible and five hidden units for randomly drawn weight and bias parameters. At these small dimensions, the probabilities associated to all possible values of the random vector **z** can be computed exactly. These probabilities are then compared to those obtained through the histogram constructed with the sampled events. To construct this histogram, each spike was extended to form a box of length τ_*r*_ (as illustrated in Figure 3), the spiking activity was sampled at 1 kHz, and the occurrences of all the possible 2^10^ states of the random vector **z** were counted. We added 1 to the number of occurrences of each state to avoid zero probabilities. The histogram obtained from a representative run is shown in Figure 4 (left).

**Figure 4:** **(Left)** Example probability distribution obtained by neural sampling of the RBM of Figure 3. The bars are marginal probabilities computed by counting the events [00000], [00001], …, [11110], [11111], respectively. *P*_NS_ is the distribution obtained by neural sampling and *P* is the exact probability distribution computed with Equation (4). **(Right)** The degree to which the sampled distribution resembles the target distribution is quantified by the KL divergence measured across 48 different distributions, and the shadings correspond to its standard deviation. This plot also shows the KL divergence of the target distribution sampled by Gibbs Sampling (*P*_Gibbs_), which is the common choice for RBMs. For comparison with the neural sampler, we identified the duration of one Gibbs sampling iteration with one refractory period τ_*r*_ = 4ms. The plot shows that up to 10^4^ms, the two methods are comparable. After this, the KL divergence of the neural sampler tends to a plateau due to the fact that neural sampling with our I&F neural network is approximate. In both figures, *P*_NS, Abstract_ refers to the marginal probability distribution obtained by using the abstract neuron model Equation (1). In this case, the KL divergence is not significantly different from the one obtained with the I&F neuron model-based sampler.

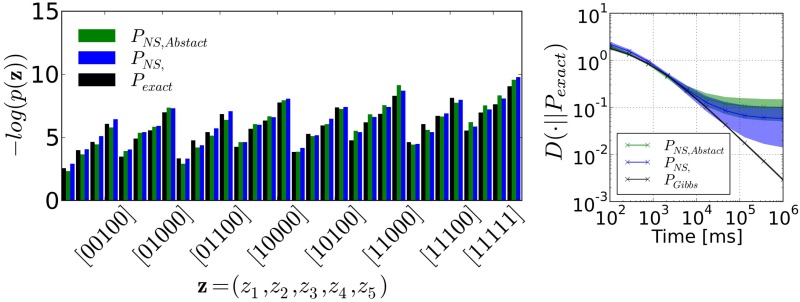



A common measure of similarity between two distributions *p* and *q* is the KL divergence:

$$D(p||q)=\sum_{i}p_{i}\log\frac{p_{i}}{q_{i}}.$$

If the distributions *p* and *q* are identical then *D*(*p*||*q*) = 0, otherwise *D*(*p*||*q*) > 0. The right panel of Figure 4 shows *D*(*p*||*P*_exact_) as a function of sampling duration, for distributions *p* obtained from three different samplers: the abstract neuron based sampler with alpha PSPs (*P*_NS,Abstract_), the I&F neuron-based sampler (*P*_NS_), and the Gibbs sampler (*P*_Gibbs_).

In the case of the I&F neuron-based sampler, the average KL divergence for 48 randomly drawn distributions after 1000s of sampling time was 0.059 ± 0.049. This result is not significantly different if the abstract neuron model Equation (1) with alpha PSPs is used (average KL divergence 0.10 ± 0.049), and in both cases the KL divergence did not tend to zero as the number of samples increased. The only difference in the latter neuron model compared to the abstract neuron model of Buesing et al. (2011), which tends to zero when sampling time tends to infinity, is the PSP model. This indicates that the discrepancy is largely due to the use of alpha-PSPs, rather than the approximation of Equation (1) with I&F neurons.

The standard sampling procedure used in RBMs is Gibbs Sampling: the neurons in the visible layer are sampled simultaneously given the activities of the hidden neurons, then the hidden neurons are sampled given the activities of the visible neurons. This procedure is iterated a number of times. For comparison with the neural sampler, the duration of one Gibbs sampling iteration is identified with one refractory period τ_*r*_ = 4ms. At this scale, we observe that the speed of convergence of the neural sampler is similar to that of the Gibbs sampler up to 10^4^ms, after which the neural sampler plateaus above the *D*(*p*||*q*) = 10^−2^ line. Despite the approximations in the neuron model and the synapse model, these results show that in RBMs of this size, the neural sampler consisting of I&F neurons sample from a distribution that has the same KL divergence as the distribution obtained after 10^4^ iterations of Gibbs sampling, which is more than the typical number of iterations used for MNIST hand-written digit tasks in the literature (Hinton et al., 2006).

### 2.3. Neural architecture for learning a model of mnist hand-written digits

**Figure 5:**
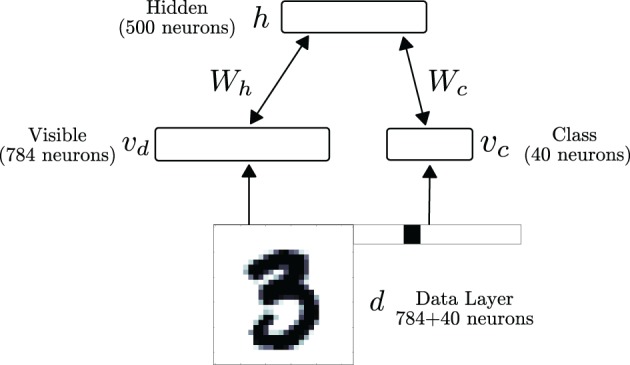
**The RBM network consists of a visible and a hidden layer**. The visible layer is partitioned into 784 sensory neurons (*v*_d_) and 40 class label neurons (*v*_c_) for supervised learning. During data presentation, the activities in the visible layer are driven by a data layer d, consisting of a digit and its label (1 neuron per label). In the RBM, the weight matrix between the visible layer and the hidden layer is symmetric.

We test the performance of the neural RBM in a digit recognition task. We use the MNIST database, whose data samples consist of centered, gray-scale, 28 × 28-pixel images of hand-written digits 0–9 (LeCun et al., 1998). The neural RBM's network architecture consisted of two layers, as illustrated in Figure 5. The visible layer was partitioned into 784 sensory neurons (*v*_d_) and 40 class label neurons (*v*_c_) for supervised learning. The pixel values of the digits were discretized to two values, with low intensity pixel values (*p* ≤ 0.5) mapped to 10^−5^ and high intensity values (*p* > 0.5) mapped to 0.98. A neuron *i* in *d* stimulated each neuron *i* in layer *v*, with synaptic currents *f*_*i*_ such that *P*(υ_*i*_ = 1) = ν(*f*_*i*_)τ_*r*_ = *p*_*i*_, where 0 ≤ *p*_*i*_ ≤ 1 is the value of pixel *i*. The value *f*_*i*_ is calculated by inverting the transfer function of the neuron: $f_{i}=\nu^{-1}(s)=\log\left(\frac{s}{\gamma -s\gamma \tau_{r}}\right)\beta^{-1}$. Using this RBM, classification is performed by choosing the most likely label given the input, under the learned model. This equals to choosing the population of class neurons associated to the same label that has the highest population firing rate.

To reconstruct a digit from a class label, the class neurons belonging to a given digit are clamped to a high firing rate. For testing the discrimination performance of an energy-based model such as the RBM, it is common to compute the free-energy *F*(**v**_**c**_) of the class units (Haykin, 1998), defined as:

(11) $$\exp(-F(v_{\text{c}}))=\sum_{v_{\text{d}},h}\exp(-E(v_{\text{d}},v_{\text{c}},h)),$$

and selecting *v*_c_ such that the free-energy is minimized. The spiking neural network is simulated using the BRIAN simulator (Goodman and Brette, 2008). All the parameters used in the simulations are provided in Table 1.

**Table 1:** **List of parameters used in the software simulations^a^**.

ν_bias_	Mean firing rate of bias Poisson spike train	All figures	1000Hz
σ	Noise amplitude	All figures, except Figure 1	3· 10^−11^ A/s^0.5^
		Figure 1 (left)	2· 10^−11^ A/s^0.5^
		Figure 1 (right)	3· 10^−10^ A/s^0.5^
		Figure 1 (bottom)	1· 10^−9^ A/s^0.5^
β	Exponential factor (fit)	All figures	2.044· 10^9^A^−1^
γ	Baseline firing rate (fit)	All figures	8808Hz
τ_*r*_	Refractory period	All figures	4ms
τ_syn_	Time constant of recurrent, and bias synapses	All figures	4ms
τ_br_	“Burn-in” time of the neural sampling	All figures	10ms
*g*_*L*_	Leak conductance	All figures	1nS
*u*_rst_	Reset potential	All figures	0V
*C*	Membrane capacitance	All figures	10^−12^F
θ	Firing threshold	All figures	100mV
*W*	RBM weight matrix (ϵ ℝ^*N*_υ_ × *N*_*h*_^)	Figure 4	*N*(−0.75, 1.5)
*b*_υ_, *b*_*h*_	RBM bias for layer υ and *h*	Figure 4	*N*(−1.5, 0.5)
*N*_υ_, *N*_*h*_	Number of visible and hidden units in the RBM	Figure 4	5,5
		Figures 7, 8, 7	824, 500
*N*_*c*_	Number of class label units	Figures 7, 8, 7	40
2*T*	Epoch duration	Figures 4, 7, 8	100ms
		Figure 9	300ms
*T*_sim_	Simulation time	Figure 2	5s
		Figure 4	1000s
		Figure 7	0.2s
		Figure 9	0.85s
		Figure 8 (testing)	1.0s
		Figure 8 (learning)	2000s
τ_STDP_	Learning time window	Figure 7	4ms
η	Learning rate	Standard CD	0.1· 10^−2^
		Event-driven CD	3.2· 10^−2^

a Software simulation scripts are available online (https://github.com/eneftci/eCD.

## 3. Results

### 3.1. Event-driven contrastive divergence

A Restricted Boltzmann Machine (RBM) is a stochastic neural network consisting of two symmetrically interconnected layers composed of neuron-like units—a set of visible units *v* and a set of hidden units *h*, but has no connections within a layer.

**Figure 6:**
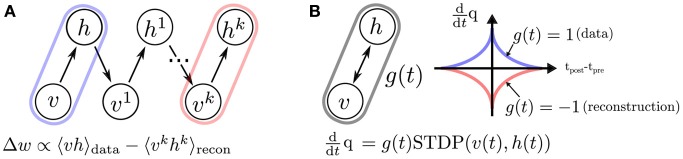
**The standard (CD)_*k*_ procedure, compared to event-driven CD**. **(A)** In standard CD, learning proceeds iteratively by sampling in “construction” and “reconstruction” phases (Hinton, 2002), which is impractical in a continuous-time dynamical system. **(B)** We propose a spiking neural sampling architecture that folds these updates on a continuous time dimension through the recurrent activity of the network. The synaptic weight update follows a STDP rule modulated by a zero mean signal *g*(*t*). This signal switches the behavior of the synapse from LTP to LTD, and partitions the training into two phases analogous to those of the original CD rule. The spikes cause microscopic weight modifications, which on average behave as the macroscopic CD weight update. For this reason, the learning rule is referred to as event-driven CD.

The training of RBMs commonly proceeds in two phases. At first the states of the visible units are clamped to a given vector from the training set, then the states of the hidden units are sampled. In a second “reconstruction” phase, the network is allowed to run freely. Using the statistics collected during sampling, the weights are updated in a way that they maximize the likelihood of the data (Hinton, 2002). Collecting equilibrium statistics over the data distribution in the reconstruction phase is often computationally prohibitive. The CD algorithm has been proposed to mitigate this (Hinton, 2002; Hinton and Salakhutdinov, 2006): the reconstruction of the visible units' activity is achieved by sampling them conditioned on the values of the hidden units (Figure 6). This procedure can be repeated *k* times (the rule is then called CD_*k*_), but relatively good convergence is obtained for the equilibrium distribution even for one iteration. The CD learning rule is summarized as follows:

(12) $$\Delta w_{ij}=\epsilon ({\langle \;\upsilon_{i}h_{j}\rangle }_{\text{data}}-{\langle \;\upsilon_{i}h_{j}\rangle }_{\text{recon}}),$$

where υ_*i*_ and *h*_*j*_ are the activities in the visible and hidden layers, respectively. This rule can be interpreted as a difference of Hebbian and anti-Hebbian learning rules between the visible and hidden neurons sampled in the data and reconstruction phases. In practice, when the data set is very large, weight updates are calculated using a subset of data samples, or “mini-batches.” The above rule can then be interpreted as a stochastic gradient descent (Robbins and Monro, 1951). Although the convergence properties of the CD rule are the subject of continuing investigation, extensive software simulations show that the rule often converges to very good solutions (Hinton, 2002).

The main result of this paper is an online variation of the CD rule for implementation in neuromorphic hardware. By virtue of neural sampling the spikes generated from the visible and hidden units can be used to compute the statistics of the probability distributions online (further details on neural sampling in the Materials and Methods section 2.1). Therefore a possible neural mechanism for implementing CD is to use synapses whose weights are governed by synaptic plasticity. Because the spikes cause the weight to update in an online, and asynchronous fashion, we refer to this rule as *event-driven* CD.

The weight update in event-driven CD is a modulated, pair-based STDP rule:

(13) $$\frac{\text{d}}{\text{d}t}q_{ij}=g(t){\text{STDP}}_{ij}(\upsilon_{i}(t),h_{j}(t))$$

where *g*(*t*) ϵ ℝ is a zero-mean global gating signal controlling the data vs. reconstruction phase, *q*_*ij*_ is the weight of the synapse and υ_*i*_(*t*) and *h*_*j*_(*t*) refer to the spike trains of neurons υ_*i*_ and *h*_*j*_, defined as in Equation (7).

As opposed to the standard CD rule, weights are updated after every occurrence of a pre-synaptic and post-synaptic event. While this online approach slightly differentiates it from standard CD, it is integral to a spiking neuromorphic framework where the data samples and weight updates cannot be stored. The weight update is governed by a symmetric STDP rule with a symmetric temporal window *K*(*t*) = *K*(−*t*), ∀*t*:

(14) $$\begin{array}{l} {\text{STDP}}_{ij}(\upsilon_{i}(t),h_{j}(t))=\upsilon_{i}(t)A_{h_{j}}(t)+h_{j}(t)A_{\upsilon_{i}}(t),\\ \;A_{h_{j}}(t)=A\int_{-\infty }^{t}\text{d}sK(t-s)h_{j}(s),\\ \;A_{\upsilon_{i}}(t)=A\int_{-\infty }^{t}\text{d}sK(s-t)\;\upsilon_{i}(s), \end{array}$$

with *A* > 0 defining the magnitude of the weight updates. In our implementation, updates are additive and weights can change polarity.

#### 3.1.1. Pairwise STDP with a global modulatory signal approximates CD

The modulatory signal *g*(*t*) switches the behavior of the synapse from LTP to LTD (i.e., Hebbian to Anti-Hebbian). The temporal average of *g*(*t*) must vanish to balance LTP and LTD, and must vary on much slower time scales than the typical times scale of the network dynamics, denoted τ_br_, so that the network samples from its stationary distribution when the weights are updated. The time constant τ_br_ corresponds to a “burn-in” time of MCMC sampling and depends on the overall network dynamics and cannot be computed in the general case. However, it is reasonable to assume τ_br_ to be in the order of a few refractory periods of the neurons (Buesing et al., 2011). In this work, we used the following modulation function *g*(*t*):

(15) $$g(t)=\{\begin{array}{ll} 1 & \text{if mod}(t,2T)\in (\tau_{\text{br}},T)\\ -1 & \text{if mod}(t,2T)\in (T+\tau_{\text{br}},2T)\\ 0 & \text{otherwise} \end{array},$$

where mod is the modulo function and *T* is a time interval. The data is presented during the time intervals (2*iT*, (2*i* + 1)*T*), where *i* is a positive integer. With the *g*(*t*) defined above, no weight update is undertaken during a fixed period τ_br_. This allows us to neglect the transients after the stimulus is turned on and off (respectively in the beginning of the data and reconstruction phases). In this case and under further assumptions discussed below, the event-driven CD rule can be directly compared with standard CD as we now demonstrate. The average weight update during (0, 2*T*) is:

(16) $$\begin{array}{l} {\langle \frac{\text{d}}{\text{d}t}q_{ij}\rangle }_{\left(0,2T\right)}=C_{ij}+R_{ij},\\ \;C_{ij}=\frac{T-\tau_{\text{br}}}{2T}({\langle \upsilon_{i}(t)A_{h_{j}}(t)\rangle }_{t_{d}}+{\langle h_{j}(t)A_{\upsilon_{i}}(t)\rangle }_{t_{d}})\\ \;R_{ij}=-\frac{T-\tau_{\text{br}}}{2T}({\langle \;\upsilon_{i}(t)A_{h_{j}}(t)\rangle }_{t_{r}}+{\langle h_{j}(t)A_{\upsilon_{i}}(t)\rangle }_{t_{r}}), \end{array}$$

where *t*_*d*_ = (τ_br_, *T*) and *t*_*r*_ = (T + τ_br_, 2*T*) denote the intervals during the positive and negative phases of *g*(*t*), and 〈 · 〉_(*a,b*)_ = $\frac{1}{b-a}\int_{a}^{b}$ d*t*·.

We write the first average in *C*_*ij*_ as follows:

(17) $$\begin{array}{l} {\langle \upsilon_{i}(t)A_{h_{j}}(t)\rangle }_{t_{d}}=A\frac{1}{T-\tau_{\text{br}}}\int_{\tau_{\text{br}}}^{T}\text{d}t\int_{-\infty }^{t}\text{d}sK(t-s)\upsilon_{i}(t)h_{j}(s),\\ \;=A\frac{1}{T-\tau_{\text{br}}}\int_{\tau_{\text{br}}}^{T}\text{d}t\int_{0}^{\infty }\text{d}\Delta K(\Delta )\upsilon_{i}(t)h_{j}(t-\Delta ),\\ \;=A\int_{0}^{\infty }\text{d}\Delta K(\Delta ){\langle \;\upsilon_{i}(t)h_{j}(t-\Delta )\rangle }_{t_{d}}. \end{array}$$

If the spike times are uncorrelated the temporal averages become a product of the average firing rates of a pair of visible and hidden neurons (Gerstner and Kistler, 2002):

$${\langle \;\upsilon_{i}(t)h_{j}(t-\Delta )\rangle }_{t_{d}}={\langle \;\upsilon_{i}(t)\rangle }_{t_{d}}{\langle h_{j}(t-\Delta )\rangle }_{t_{d}}=:{\bar{\upsilon }}_{i}^{+}{\bar{h}}_{j}^{+}.$$

If we choose a temporal window that is much smaller than *T*, and assume the network activity is stationary in the interval (τ_br_, *T*), we can write (up to a negligible error Kempter et al., 2001)

(18) $${\langle \upsilon_{i}(t)A_{h_{j}}(t)\rangle }_{t_{d}}=A{\bar{\upsilon }}_{i}^{+}{\bar{h}}_{j}^{+}\int_{0}^{\infty }\text{d}\Delta K(\Delta ).$$

In the uncorrelated case, the second term in *C*_*ij*_ contributes the same amount, leading to:

$$C_{ij}=\eta {\bar{\upsilon }}_{i}^{+}{\bar{h}}_{j}^{+}.$$

with $\eta \;=:2A\frac{T-\tau_{\text{br}}}{2T}\int_{0}^{\infty }\text{d}\Delta K(\Delta )$. Similar arguments apply to the averages in the time interval *t*_*r*_:

$$R_{ij}=2A\int_{0}^{\infty }\text{d}\Delta K(\Delta ){\langle \upsilon_{i}(t)h_{j}(t-\Delta )\rangle }_{t_{r}}=\eta {\bar{\upsilon }}_{i}^{-}{\bar{h}}_{j}^{-}.$$

with υ^−^_*i*_ *h*^−^_*j*_ := 〈υ_*i*_(*t*)〉_*t*_r__〈*h*_*j*_(*t* − Δ)〉_*t*_*r*__. The average update in (0, 2*T*) then becomes:

(19) $${\langle \frac{\text{d}}{\text{d}t}q_{ij}\rangle }_{\left(0,2T\right)}=\eta \left({\bar{\upsilon }}_{i}^{+}{\bar{h}}_{j}^{+}-{\bar{\upsilon }}_{i}^{-}{\bar{h}}_{j}^{-}\right).$$

According to Equation (18), any symmetric temporal window that is much shorter than *T* can be used. For simplicity, we choose an exponential temporal window *K*(Δ) = exp(−|Δ/τ_STDP_|) with decay rate τ_STDP_ ≪ *T* (Figure 6B). In this case, $\eta =2A\frac{T-\tau_{\text{br}}}{2T}\tau_{\text{STDP}}$.

The modulatory function *g*(*t*) partitions the training into epochs of duration 2*T*. Each epoch consists of a LTP phase during which the data is presented (construction), followed by a free-running LTD phase (reconstruction). The weights are updated asynchronously during the time interval in which the neural sampling proceeds, and Equation (19) tells us that its average resembles Equation (12). However, it is different in two ways: the averages are taken over one data and reconstruction phase rather than a mini-batch of data samples and their reconstructions; and more importantly, the synaptic weights are updated during the data and the reconstruction phase, whereas in the CD rule, updates are carried out at the end of the reconstruction phase. In the derivation above the effect of the weight change on the network during an epoch 2*T* was neglected for mathematical simplicity. In the following, we verify that despite this approximation, the event-driven CD performs nearly as well as standard CD in the context of a common benchmark task.

### 3.2. Learning a generative model of hand-written digits

We train the RBM to learn a generative model of the MNIST handwritten digits using event-driven CD (see section 2.3 for details). For training, 20,000 digits selected randomly (with repetition) from a training set consisting of 10,000 digits were presented in sequence, with an equal number of samples for each digit.

**Figure 7:**
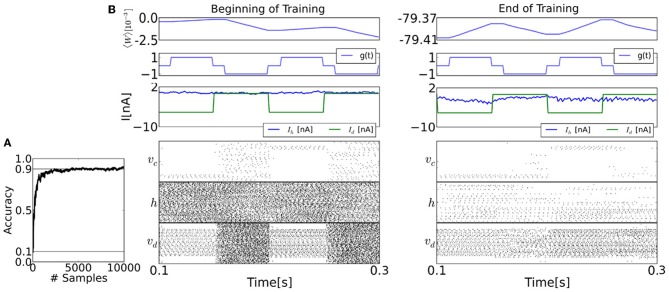
**The spiking neural network learns a generative model of the MNIST dataset using the event-driven CD procedure**. **(A)** Learning curve, shown here up to 10,000 samples. **(B)** Details of the training procedure, before and after training (20,000 samples). During the first half of each 0.1s epoch, the visible layer *v* is driven by the sensory layer, and the gating variable *g* is 1, meaning that the synapses undergo LTP. During the second half of each epoch, the sensory stimulus is removed, and *g* is set to −1, so the synapses undergo LTD. The top panels of both figures show the mean of the entries of the weight matrix. The second panel shows the values of the modulatory signal *g*(*t*). The third panel shows the synaptic currents of a visible neuron, where *I*_*h*_ is caused by the feedback from the hidden and the bias, and *I*_*d*_ is the data. The timing of the clamping (*I*_*d*_) and *g* differ due to an interval τ_br_ where no weight update is undertaken to avoid the transients (see section 2). Before learning and during the reconstruction phase, the activity of the visible layer is random. But as learning progresses, the activity in the visible layer reflects the presented data in the reconstruction phase. This is very well visible in the layer class label neurons *v*_*c*_, whose activity persists after the sensory stimulus is removed. Although the firing rates of the hidden layer neurons before training is high (average 113Hz), this is only a reflection of the initial conditions for the recurrent couplings *W*. In fact, at the end of the training, the firing rates in both layers becomes much sparser (average 9.31Hz).

The raster plots in Figure 7 show the spiking activity of each layer before and after learning for epochs of duration 100ms. The top panel shows the population-averaged weight. After training, the sum of the upwards and downward excursions of the average weight is much smaller than before training, because the learning is near convergence. The second panel shows the value of the modulatory signal *g*(*t*). The third panel shows the input current (*I*_*d*_) and the current caused by the recurrent couplings (*I*_*h*_).

**Figure 8:**
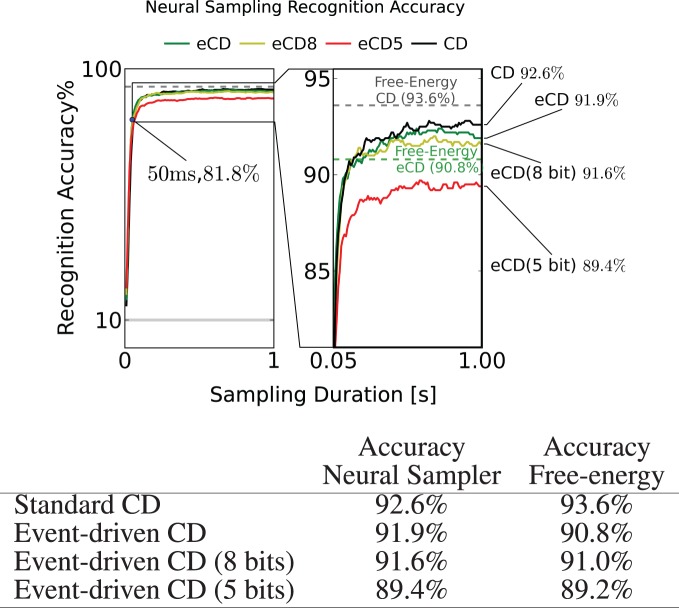
**To test recognition accuracy, the trained RBMs are sampled using the I&F neuron-based sampler for up to 1s**. The classification is read out by identifying the group of class label neurons that had the highest activity. This experiment is run for RBM parameter sets obtained by standard CD (black, CD) and event-driven CD (green, eCD). To test for robustness to finite precision weights, the RBM was run with parameters obtained by event-driven CD discretized to 8 and 5 bits. In all scenarios, the accuracy after 50ms of sampling was above 80% and after 1s the accuracies typically reached their peak at around 92%. The dashed horizontal lines show the recognition accuracy obtained by minimizing the free-energy (see text). The fact that the eCD curve (solid green) surpasses its free-energy line suggests that a model that is tailored to the I&F spiking neural network was learned.

Two methods can be used to estimate the overall recognition accuracy of the neural RBM. The first is to sample: the visible layer is clamped to the digit only (i.e., υ_*d*_), and the network is run for 1s. The known label is then compared with the position of the group of class neurons that fired at the highest rate. The second method is to minimize free-energy: the neural RBMs parameters are extracted, and for each data sample, the class label with the lowest free-energy (see section 2) is compared with the known label. In both cases, recognition was tested for 1000 data samples that were not used during the training. The results are summarized in Figure 8.

As a reference we provide the best performance achieved using the standard CD and one unit per class label (*N*_*c*_ = 10) (Figure 8, table row 1), 93.6%. By mapping the these parameters to the neural sampler, the recognition accuracy reached 92.6%. The discrepancy is expected since the neural sampler does not exactly sample from the target Boltzmann distribution (see section 2.2).

When training a neural RBM of I&F neurons using event-driven CD, the recognition result was 91.9% (Figure 8, table row 2). The performance of this RBM obtained by minimizing its free-energy was 90.8%. The learned parameters performed well for classification using the free-energy calculation which suggests that the network learned a model that is consistent with the mathematical description of the RBM.

In an energy-based model like the RBM the free-energy minimization should give the upper bound on the discrimination performance (Haykin, 1998). For this reason, the fact that the recognition accuracy is higher when sampling as opposed to using the free-energy method may appear puzzling. However, this is possible because the neural RBM does not exactly sample from the Boltzmann distribution, as explained in section 2.2. This suggests that event-driven CD compensates for the discrepancy between the distribution sampled by the neural RBM and the Boltzmann distribution, by learning a model that is tailored to the spiking neural network.

Excessively long training durations can be impractical for real-time neuromorphic systems. Fortunately, the learning using event-driven CD is fast: Compared to the off-line RBM training (250, 000 presentations, in mini-batches of 100 samples) the event-driven CD training succeeded with a smaller number of data presentations (20, 000), which corresponded to 2000s of simulated time. This suggests that the training durations are achievable for real-time neuromorphic systems.

#### 3.2.1. The choice of the number of class neurons *N*_*c*_

Event-driven CD underperformed in the case of 1 neuron per class label (*N*_*c*_ = 10), which is the common choice for standard CD and Gibbs sampling. This is because a single neuron firing at its maximum rate of 250 Hz cannot efficiently drive the rest of the network without tending to induce spike-to-spike correlations (e.g., synchrony), which is incompatible with the assumptions made for sampling with I&F neurons and event-driven CD. As a consequence, the generative properties of the neural RBM degrade. This problem is avoided by using several neurons per class label (in our case four neurons per class label) because the synaptic weight can be much lower to achieve the same effect, resulting in smaller spike-to-spike correlations.

#### 3.2.2. Neural parameters with finite precision

In hardware systems, the parameters related to the weights and biases cannot be set with floating-point precision, as can be done in a digital computer. In current neuromorphic implementations the synaptic weights can be configured at precisions of about 8 bits (Yu et al., 2012). We characterize the impact of finite-precision synaptic weights on performance by discretizing the weight and bias parameters to 8 bits and 5 bits. The set of possible weights were spaced uniformly in the interval (μ −4.5σ, μ + 4.5 σ), where μ,σ are the mean and the standard deviation of the parameters across the network, respectively. The classification performance of MNIST digits degraded gracefully. In the 8 bit case, it degrades only slightly to 91.6%, but in the case of 5 bits, it degrades more substantially to 89.4%. In both cases, the RBM still retains its discriminative power, which is encouraging for implementation in hardware neuromorphic systems.

### 3.3. Generative properties of the RBM

**Figure 9:**
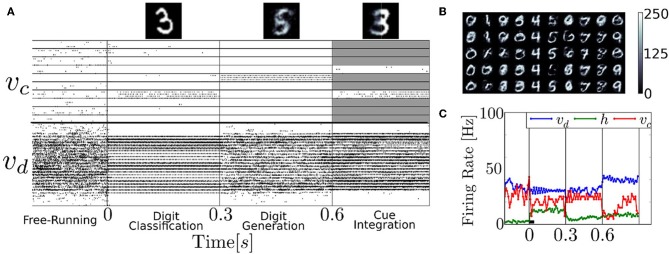
**The recurrent structure of the network allows it to classify, reconstruct and infer from partial evidence**. **(A)** Raster plot of an experiment illustrating these features. Before time 0s, the neural RBM runs freely, with no input. Due to the stochasticity in the network, the activity wanders from attractor to attractor. At time 0s, the digit 3 is presented (i.e., layer υ_*d*_ is driven by *d*), activating the correct class label in υ_*c*_; At time *t* = 0.3 s, the class neurons associated to 5 are clamped to high activity and the rest of the class label neurons are strongly inhibited, driving the network to reconstruct its version of the digit in layer υ_*d*_; At time *t* = 0.6 s, the right-half part of a digit 8 is presented, and the class neurons are stimulated such that only 3 or 6 can activate (all others are strongly inhibited as indicated by the gray shading). Because the stimulus is inconsistent with 6, the network settles to a 3 and attempts to reconstruct it. The top figures show the digits reconstructed in layer υ_*d*_. **(B)** Digits 0–9, reconstructed in the same manner. The columns correspond to clamping digits 0–9, and each is different, independent run. **(C)** Population firing rate of the experiment presented in **(A)**. The network activity is typically at equilibrium after about 10τ_*r*_ = 40ms (black bar).

We test the neural RBM as a generative model of the MNIST dataset of handwritten digits, using parameters obtained by running the event-driven CD. The RBM's generative property enables it to classify and generate digits, as well as to infer digits by combining partial evidence. These features are clearly illustrated in the following experiment (Figure 9). First the digit 3 is presented (i.e., layer υ_*d*_ is driven by layer d) and the correct class label in *v*_*c*_ activated. Second, the neurons associated to class label 5 are clamped, and the network generated its learned version of the digit. Third, the right-half part of a digit 8 is presented, and the class neurons are stimulated such that only 3 or 6 are able to activate (the other class neurons are inhibited, indicated by the gray shading). Because the stimulus is inconsistent with 6, the network settled to 3 and reconstructed the left part of the digit.

The latter part of the experiment illustrates the integration of information between several partially specified cues, which is of interest for solving sensorimotor transformation or multi-modal sensory cue integration problems (Deneve et al., 2001; Doya et al., 2006; Corneil et al., 2012). This feature has been used for auditory-visual sensory fusion in a spiking Deep Belief Network (DBN) model (O'Connor et al., 2013). There, the authors trained a DBN with visual and auditory data, which learned to associate the two sensory modalities, very similarly to how class labels and visual data are associated in our architecture. Their network was able to resolve a similar ambiguity as in our experiment in Figure 9, but using auditory inputs instead of a class label.

During digit generation, the trained network had a tendency to be globally bistable, whereby the layer υ_*d*_ completely deactivated layer *h*. Since all the interactions between υ_*d*_ and υ_*c*_ take place through the hidden layer, υ_*c*_ could not reconstruct the digit. To avoid this, we added populations of I&F neurons that were wired to layers υ and *h*, respectively. The parameters of these neurons and their couplings were tuned such that each layer was strongly excited when it's average firing rate fell below 5Hz.

## 4. Discussion

Neuromorphic systems are promising alternatives for large-scale implementations of RBMs and deep networks, but the common procedure used to train such networks, (CD), involves iterative, discrete-time updates that do not straightforwardly map on a neural substrate. We solve this problem in the context of the RBM with a spiking neural network model that uses the recurrent network dynamics to compute these updates in a continuous-time fashion. We argue that the recurrent activity coupled with STDP dynamics implements an event-driven variant of CD. Event-driven CD enables the system to learn on-line, while being able to carry out functionally relevant tasks such as recognition, data generation and cue integration.

The CD algorithm can be used to learn the parameters of probability distributions other than the Boltzmann distribution (even those without any symmetry assumptions). Our choice for the RBM, whose underlying probability distribution is a special case of the Boltzmann distribution, is motivated by the following facts: They are universal approximators of discrete distributions (Le Roux and Bengio, 2008); the conditions under which a spiking neural circuit can naturally perform MCMC sampling of a Boltzmann distribution were previously studied (Merolla et al., 2010; Buesing et al., 2011); and RBMs form the building blocks of many deep learning models such as DBNs, which achieve state-of-the-art performance in many machine learning tasks (Bengio, 2009). The ability to implement RBMs with spiking neurons and train then using event-based CD paves the way toward on-line training of DBNs of spiking neurons (Hinton et al., 2006).

We chose the MNIST handwritten digit task as a benchmark for testing our model. When the RBM was trained with standard CD, it could recognize up to 926 out of 1000 of out-of-training samples. The MNIST handwritten digit recognition task was previously shown in a digital neuromorphic chip (Arthur et al., 2012), which performed at 89% accuracy, and in a software simulated visual cortex model (Eliasmith et al., 2012). However, both implementations were configured using weights trained off-line. A recent article showed the mapping of off-line trained DBNs onto spiking neural network (O'Connor et al., 2013). Their results demonstrated hand-written digit recognition using neuromorphic event-based sensors as a source of input spikes. Their performance reached up to 94.1% using leaky I&F neurons. The use of an additional layer explains to a large extent their better performance compared to ours (91.9%). Our work extends (O'Connor et al., 2013) with on-line training that is based on synaptic plasticity, testing its robustness to finite weight precision, and providing an interpretation of spiking activity in terms of neural sampling.

To achieve the computations necessary for sampling from the RBM, we have used a neural sampling framework (Fiser et al., 2010), where each spike is interpreted as a sample of an underlying probability distribution. Buesing et al. proved that abstract neuron models consistent with the behavior of biological spiking neurons can perform MCMC, and have applied it to a basic learning task in a fully visible Boltzmann Machine. We extended the neural sampling framework in three ways: First, we identified the conditions under which a dynamical system consisting of I&F neurons can perform neural sampling; Second, we verified that the sampling of RBMs was robust to finite-precision parameters; Third, we demonstrated learning in a Boltzmann Machine with hidden units using STDP synapses.

In neural sampling, neurons behave stochastically. This behavior can be achieved in I&F neurons using noisy input currents, created by a Poisson spike train. Spike trains with Poisson-like statistics can be generated with no additional source of noise, for example by the following mechanisms: balanced excitatory and inhibitory connections (van Vreeswijk and Sompolinsky, 1996), finite-size effects in a large network, and neural mismatch (Amit and Brunel, 1997). The latter mechanism is particularly appealing, because it benefits from fabrication mismatch and operating noise inherent to neuromorphic implementations (Chicca and Fusi, 2001).

Other groups have also proposed to use I&F neuron models for computing the Boltzmann distribution. (Merolla et al., 2010) have shown that noisy I&F neurons' activation function is approximately a sigmoid as required by the Boltzmann machine, and have devised a scheme whereby a global inhibitory rhythm drives the network to generate samples of the Boltzmann distribution. O'Connor et al. (2013) have demonstrated a deep belief network of I&F neurons that was trained off-line, using standard CD and tested it using the MNIST database. Independently and simultaneously to this work, Petrovici et al. (2013) demonstrated that conductance-based I&F neurons in a noisy environment are compatible with neural sampling as described in Buesing et al. (2011). Similarly, Petrovici et al. find that the choice of non-rectangular PSPs and the approximations made by the I&F neurons are not critical to the performance of the neural sampler. Our work extends all of those above by providing an online, STDP-based learning rule to train RBMs sampled using I&F neurons.

### 4.1. Applicability to neuromorphic hardware

Neuromorphic systems are sensible to fabrication mismatch and operating noise. Fortunately, the mismatch in the synaptic weights and the activation function parameters γ and β are not an issue if the biases and the weights are learned, and the functionality of the RBM is robust to small variations in the weights caused by discretization. These two findings are encouraging for neuromorphic implementations of RBMs. However, at least two conceptual problems of the presented RBM architecture must be solved in order to implement such systems on a larger scale. First, the symmetry condition required by the RBM does not necessarily hold. In a neuromorphic device, the symmetry condition is impossible to guarantee if the synapse weights are stored locally at each neuron. Sharing one synapse circuit per pair of neurons can solve this problem. This may be impractical due to the very large number of synapse circuits in the network, but may be less problematic when using Resistive Random-Access Memorys (RRAMs) (also called *memristors*) crossbar arrays to emulate synapses (Kuzum et al., 2011; Cruz-Albrecht et al., 2013; Serrano-Gotarredona et al., 2013). RRAM are a new class of nanoscale devices whose current-voltage relationship depends on the history of other electrical quantities (Strukov et al., 2008), and so act like programmable resistors. Because they can conduct currents in both directions, one RRAM circuit can be shared between a pair of neurons. A second problem is the number of recurrent connections. Even our RBM of modest dimensions involved almost two million synapses, which is impractical in terms of bandwidth and weight storage. Even if a very high number of weights are zero, the connections between each pair of neurons must exist in order for a synapse to learn such weights. One possible solution is to impose sparse connectivity between the layers (Murray and Kreutz-Delgado, 2007; Tang and Eliasmith, 2010) and implement synaptic connectivity in a scalable hierarchical address-event routing architecture (Joshi et al., 2010; Park et al., 2012).

### 4.2. Outlook: a custom learning rule

our method combines I&F neurons that perform neural sampling and the CD rule. although we showed that this leads to a functional model, we do not know whether event-driven CD is optimal in any sense. This is partly due to the fact that CD_*k*_ is an approximate rule (Hinton, 2002), and it is still not entirely understood why it performs so well, despite extensive work in studying its convergence properties (Carreira-Perpinan and Hinton, 2005). furthermore, the distribution sampled by the I&F neuron does not exactly correspond to the Boltzmann distribution and the average weight updates in event-driven CD differ from those of standard CD, because in the latter they are carried out at the end of the reconstruction step.

A very attractive alternative is to derive a custom synaptic plasticity rule that minimizes some functionally relevant quantity (such as Kullback-Leibler divergence or Contrastive Divergence), *given* the encoding of the information in the I&F neuron (Deneve, 2008; Brea et al., 2013). A similar idea was recently pursued in Brea et al. (2013), where the authors derived a triplet-based synaptic learning rule that minimizes an upper bound of the Kullback–Leibler divergence between the model and the data distributions. Interestingly, their rule had a similar global signal that modulates the learning rule, as in event-driven CD, although the nature of this resemblance remains to be explored. Such custom learning rules can be very beneficial in guiding the design of on-chip plasticity in neuromorphic VLSI and RRAM nanotechnologies, and will be the focus of future research.

### Conflict of interest statement

The authors declare that the research was conducted in the absence of any commercial or financial relationships that could be construed as a potential conflict of interest.
